# Supplementary material for: Contextualizing goal preferences in fear-avoidance models. Looking at fatigue as a disabling symptom in fibromyalgia patients
Source: PLoS One. 2021 Jul 8;16(7):e0254200. doi: 10.1371/journal.pone.0254200 (PMC8266041; doi:10.1371/journal.pone.0254200)
Supplement: S1 File — (PDF) [file pone.0254200.s001.pdf]

Goal Pursuit Questionnaire Fatiga (GPQ-F)

A continuación se describen varias situaciones que le pueden ocurrir en su vida cotidiana. Lea cada una atentamente e intente ponerse en el lugar de esa situación lo mejor que pueda. Son situaciones hipotéticas. Es posible que no las haya vivido nunca o no las vaya a vivir (por ejemplo, no trabaja o no usa el ordenador). Le pedimos que conteste imaginándose que está en esa situación.
Cada situación va seguida de un pensamiento que una persona puede tener en ella. Por eso, después se le pide que indique su grado de acuerdo con ese pensamiento, rodeando con un círculo un número del 1 al 6. Cuanto mayor sea el número, más de acuerdo estará usted con ese pensamiento. **Preste atención, la cuestión no es lo que usted haría, sino lo que usted pensaría en esa situación.**

Situación 2: Usted está contando sus emocionantes vacaciones o alguna cosa emocionante que le ha pasado. Al contarlo, cada vez le queda menos tiempo para la montaña de trabajo que le está esperando. Se espera que hoy sin falta termine todo ese trabajo

Usted piensa: Creo que es más importante contar mis vacaciones o algo emocionante que me ha pasado que terminar mi trabajo.

¿En qué medida está de acuerdo con este pensamiento?:

Completamente en desacuerdo	Bastante en desacuerdo	Un poco en desacuerdo	Un poco de acuerdo	Bastante de acuerdo	Completamente de acuerdo
1	2	3	4	5	6

Situación 3: Usted está limpiando los cristales de las ventanas. A medida que limpia, va teniendo fatiga. Se espera que hoy termine de limpiar los cristales.

Usted piensa: Creo que es más importante que disminuya ahora la fatiga que acabar de limpiar los cristales.

¿En qué medida está de acuerdo con este pensamiento?:

Completamente en desacuerdo	Bastante en desacuerdo	Un poco en desacuerdo	Un poco de acuerdo	Bastante de acuerdo	Completamente de acuerdo
1	2	3	4	5	6

Situación 4: Usted comienza a leer un libro muy interesante. Al leer el libro le va quedando cada vez menos tiempo para terminar un informe. Su jefe espera que termine hoy su informe.

Usted piensa: Creo que es más importante leer ahora este libro tan interesante que terminar de escribir el informe a tiempo.

¿En qué medida está de acuerdo con este pensamiento?:

Completamente en desacuerdo	Bastante en desacuerdo	Un poco en desacuerdo	Un poco de acuerdo	Bastante de acuerdo	Completamente de acuerdo
1	2	3	4	5	6

Situación 6:	Usted está cargando las bolsas de la compra. Después de un rato cargándolas, cada vez tiene más fatiga. Se espera que hoy termine de hacer las compras.
Usted piensa:	Creo que es más importante que mi fatiga disminuya ahora, que terminar de hacer las compras.

¿En qué medida está de acuerdo con este pensamiento?:

Completamente en desacuerdo 1	Bastante en desacuerdo 2	Un poco en desacuerdo 3	Un poco de acuerdo 4	Bastante de acuerdo 5	Completamente de acuerdo 6

Situación 7:	Usted está pasando la aspiradora por la casa. Al hacerlo, cada vez tiene más fatiga. Se espera que hoy acabe de limpiar la casa.
Usted piensa:	Creo que es más importante que mi fatiga disminuya ahora, que terminar de limpiar la casa.

¿En qué medida está de acuerdo con este pensamiento?:

Completamente en desacuerdo 1	Bastante en desacuerdo 2	Un poco en desacuerdo 3	Un poco de acuerdo 4	Bastante de acuerdo 5	Completamente de acuerdo 6

Situación 9:	Usted está organizando la ropa para poner la lavadora. Como es un trabajo monótono, se aburre cada vez más. Se espera que hoy termine este trabajo.
Usted piensa:	Creo que es más importante quitarme el aburrimiento que terminar este trabajo.

¿En qué medida está de acuerdo con este pensamiento?:

Completamente en desacuerdo 1	Bastante en desacuerdo 2	Un poco en desacuerdo 3	Un poco de acuerdo 4	Bastante de acuerdo 5	Completamente de acuerdo 6

Situación 10:	Usted recibe un correo electrónico o un WhatsApp divertido. Debido a que quiere responder, cada vez le queda menos tiempo para realizar una tarea. Se espera que hoy termine de realizarla.
Usted piensa:	Creo que es más importante responder con un mensaje divertido que terminar la tarea.

¿En qué medida está de acuerdo con este pensamiento?:

Completamente en desacuerdo 1	Bastante en desacuerdo 2	Un poco en desacuerdo 3	Un poco de acuerdo 4	Bastante de acuerdo 5	Completamente de acuerdo 6

Situación 11:	Usted está haciendo un álbum de fotos a mano o en el ordenador. Al hacer este trabajo, cada vez tiene más fatiga. Se espera que hoy termine el álbum.
Usted piensa:	Creo que es más importante que disminuya ahora mi fatiga que terminar el álbum.
¿En qué medida está de acuerdo con este pensamiento?:	

Completamente en desacuerdo	Bastante en desacuerdo	Un poco en desacuerdo	Un poco de acuerdo	Bastante de acuerdo	Completamente de acuerdo
1	2	3	4	5	6

Situación 12:	Usted tiene que hacer varias tareas de costura. De tanto coser siente cada vez más fatiga. Se espera que hoy siga cosiendo hasta que termine la costura.
Usted piensa:	Creo que es más importante que la fatiga disminuya ahora que terminar la costura.
¿En qué medida está de acuerdo con este pensamiento?:	

Completamente en desacuerdo	Bastante en desacuerdo	Un poco en desacuerdo	Un poco de acuerdo	Bastante de acuerdo	Completamente de acuerdo
1	2	3	4	5	6

Situación 13:	Usted tiene una agradable conversación durante una consulta profesional. Debido a la conversación, cada vez tienen menos tiempo para tomar una serie de decisiones. Se espera que las decisiones se tomen hoy.
Usted piensa:	Creo que es más importante tener esta agradable conversación en la que estoy ahora que terminar de tomar las decisiones.
¿En qué medida está de acuerdo con este pensamiento?:	

Completamente en desacuerdo	Bastante en desacuerdo	Un poco en desacuerdo	Un poco de acuerdo	Bastante de acuerdo	Completamente de acuerdo
1	2	3	4	5	6

Situación 14:	Está limpiando el coche. Al utilizar los utensilios de limpieza cada vez siente más fatiga. Se espera que hoy termine de limpiar el coche.
Usted piensa:	Creo que es más importante que la fatiga disminuya ahora, a que el coche esté limpio.
¿En qué medida está de acuerdo con este pensamiento?:	

Completamente en desacuerdo	Bastante en desacuerdo	Un poco en desacuerdo	Un poco de acuerdo	Bastante de acuerdo	Completamente de acuerdo
1	2	3	4	5	6

Situación 15:	Está disfrutando mientras que ve un programa de televisión. Por eso casi se olvida de que hoy tiene otras tareas que hacer. Se espera que termine también sus tareas.
Usted piensa:	Creo que es más importante pasarlo bien ahora que terminar mis tareas.

¿En qué medida está de acuerdo con este pensamiento?:

Completamente en desacuerdo	Bastante en desacuerdo	Un poco en desacuerdo	Un poco de acuerdo	Bastante de acuerdo	Completamente de acuerdo
1	2	3	4	5	6

Goal Pursuit Questionnaire Fatigue (GPQ-F)

Below are several situations that may occur in your everyday life. Read each one carefully and try to put yourself in the shoes of that situation to the best of your ability. They are hypothetical situations. It is possible that you has never experienced them or will not experience them (for example, you do not work or do not use the computer). We ask you to answer by imagining that you are in that situation.
Each situation is followed by a thought that a person may have in it. Therefore, you are then asked to indicate your degree of agreement with that thought by circling a number from 1 to 6. The higher the number, the more you agree with that thought. **Pay attention, the question is not what you would do, but what you would think in that situation.**

Situation 2:	You are talking about your exciting holidays or something exciting that happened to you. By talking about it, you have less and less time for the mountain of work that awaits you. It is expected that today without fail to finish all that work.				
You think:	I think it is more important to tell my holiday stories or something amazing than to finish my work.				
To what extent do you agree with this thought ?:					
Very strongly disagree<br>1	Strongly disagree<br>2	Disagree<br>3	Agree<br>4	Strongly agree<br>5	Very strongly agree<br>6

Situation 3:	You are cleaning the windows. As you clean, you become fatigued. You are expected to finish cleaning the windows today.				
You think:	I think it is more important to reduce my fatigue now than to finish cleaning the windows.				
To what extent do you agree with this thought ?:					
Very strongly disagree<br>1	Strongly disagree<br>2	Disagree<br>3	Agree<br>4	Strongly agree<br>5	Very strongly agree<br>6

Situation 4:	You start reading a very interesting book. As you read the book, you have less and less time to finish a report. Your boss expects you to finish your report today.				
You think:	I think it is more important to read this interesting book now than to finish writing the report on time.				
To what extent do you agree with this thought ?:					
Very strongly disagree<br>1	Strongly disagree<br>2	Disagree<br>3	Agree<br>4	Strongly agree<br>5	Very strongly agree<br>6

Situation 6:	You are loading the shopping bags. After charging them for a while, you get more and more fatigued. You are expected to finish shopping today.				
You think:	I think it is more important that my fatigue reduce now than to finish shopping.				
To what extent do you agree with this thought ?:					
Very strongly disagree 1	Strongly disagree 2	Disagree 3	Agree 4	Strongly agree 5	Very strongly agree 6

Situation 7:	You are vacuuming the house. As you do so, you get more and more fatigued. You hope to finish cleaning the house today.				
You think:	I think it is more important that my fatigue reduce now than to finish cleaning the house.				
To what extent do you agree with this thought ?:					
Very strongly disagree 1	Strongly disagree 2	Disagree 3	Agree 4	Strongly agree 5	Very strongly agree 6

Situation 9:	You are organizing clothes to put in the washing machine. As it is a monotonous job, you get more and more bored. You hope to finish this work today.				
You think:	I think it is more important to get rid of boredom than to finish this job.				
To what extent do you agree with this thought ?:					
Very strongly disagree 1	Strongly disagree 2	Disagree 3	Agree 4	Strongly agree 5	Very strongly agree 6

Situation 10:	You get a funny email or whatsapp. Because you want to respond, you have less and less time to complete a task. It is expected that it will finish today.				
You think:	I think it's more important to reply with a funny message than to finish the task.				
To what extent do you agree with this thought ?:					
Very strongly disagree 1	Strongly disagree 2	Disagree 3	Agree 4	Strongly agree 5	Very strongly agree 6

Situation 11:	You are making a photo album by hand or on the computer. By doing this work, you become more and more fatigued. You hope to finish the album today.
You think:	I think it is more important to reduce my fatigue now than to finish the album.
To what extent do you agree with this thought ?:	

Very strongly disagree 1	Strongly disagree 2	Disagree 3	Agree 4	Strongly agree 5	Very strongly agree 6

Situation 12:	You have to do various sewing tasks. From so much sewing you feel more and more fatigue. Today you are expected to continue sewing until the sewing is finished.
You think:	I think it is more important that the fatigue decrease now than to finish sewing.
To what extent do you agree with this thought ?:	

Very strongly disagree 1	Strongly disagree 2	Disagree 3	Agree 4	Strongly agree 5	Very strongly agree 6

Situation 13:	You have a pleasant conversation during a professional consultation. Due to the conversation, you have less and less time to make a series of decisions. Decisions are expected to be made today.
You think:	I think it is more important to have this nice conversation that I am in now, than to finish making decisions.
To what extent do you agree with this thought ?:	

Very strongly disagree 1	Strongly disagree 2	Disagree 3	Agree 4	Strongly agree 5	Very strongly agree 6

Situation 14:	You are cleaning the car. When using cleaning utensils you feel more and more fatigued. You hope to finish cleaning the car today.
You think:	I think it is more important that the fatigue decreases now, that the car is clean.
To what extent do you agree with this thought ?:	

Very strongly disagree 1	Strongly disagree 2	Disagree 3	Agree 4	Strongly agree 5	Very strongly agree 6

Situation 15:	You are enjoying yourself while watching a television show. That's why you almost forget that today you have other tasks to do. You are expected to complete your assignments as well.
You think:	I think it's more important to have fun now than to finish my homework.

To what extent do you agree with this thought ?:

Very strongly disagree	Strongly disagree	Disagree	Agree	Strongly agree	Very strongly agree
1	2	3	4	5	6
